# Cryptic Species Discrimination in Western Pine Beetle, *Dendroctonus brevicomis* LeConte (Curculionidae: Scolytinae), Based on Morphological Characters and Geometric Morphometrics

**DOI:** 10.3390/insects10110377

**Published:** 2019-10-30

**Authors:** Osiris Valerio-Mendoza, Jazmín García-Román, Moises Becerril, Francisco Armendáriz-Toledano, Gerardo Cuéllar-Rodríguez, José F. Negrón, Brian T. Sullivan, Gerardo Zúñiga

**Affiliations:** 1 Prolongación de Carpio y Plan de Ayala, Laboratorio de Variación Biológica y Evolución, Zoología, Depto, Escuela Nacional de Ciencias Biológicas, Instituto Politécnico Nacional, Col. Santo Tomas, Del. Miguel Hidalgo, Ciudad de México CP 11340, Mexico; ovaleriom82@gmail.com (O.V.-M.); jazminjackman@hotmail.com (J.G.-R.); moises.becerril.891123@live.com (M.B.); onirica.2@hotmail.com (F.A.-T.); 2 Facultad de Ciencias Forestales, Universidad Autónoma de Nuevo León, Carretera Nacional No. 85, Km. 145, Linares, Nuevo León CP 67700, Mexico; entomolab@gmail.com; 3 United States Department of Agriculture, Forest Service, Rocky Mountain Research Station, 240 West Prospect, Fort Collins, CO 80525, USA; jnegron@fs.fed.us; 4 United States Department of Agriculture, Forest Service, Southern Research Station, 2500 Shreveport Hwy, Pineville, LA 71360, USA; briansullivan@fs.fed.us

**Keywords:** *Dendroctonus frontalis* complex, taxonomy species delimiting, morphometrics

## Abstract

The western pine beetle (WPB), *Dendroctonus brevicomis* LeConte, is a major mortality agent of pines in North America. A total of 706 adults of WPB from 81 geographical sites were analyzed with traditional and geometric morphometric methods to evaluate the variation of discrete and quantitative morphological characters with particular attention to the antenna, spermatheca, and seminal rod. Principal coordinates and canonical variate analyses supported three geographical groups in WPB: (1) West, from British Columbia to southern California along the Pacific coast, Idaho, and Montana; (2) East-SMOC, including Nevada, Utah, Colorado, Arizona, New Mexico, Texas, Chihuahua, and Durango; and (3) SMOR, including Coahuila, Nuevo Leon, and Tamaulipas. The pubescence length on the elytral declivity was a robust character for separating West specimens from the other groups. Additionally, the genitalia shape both female and male in dorsal view was a reliable character for discriminating among groups. Based on these results, which agree with genetic and chemical ecology evidence, we herein reinstate *Dendroctonus barberi* Hopkins (East-SMOC group) and remove it from synonymy with *D. brevicomis* (West group). Differences in the spermatheca and seminal rod shape of SMOR specimens suggest that these populations might be a different species from *D. barberi* and *D. brevicomis*.

## 1. Introduction

The western pine beetle, *Dendroctonus brevicomis* LeConte, is a North American species distributed along the Pacific coast from southern British Columbia to southern California, in Idaho and eastern Montana, and from Nevada, Utah, and Colorado southeast to Durango and Tamaulipas [1,2]. *Dendroctonus brevicomis* is a facultative predator that, during outbreaks in Canada and the United States, colonizes and kills millions of trees, mainly ponderosa pine (*Pinus ponderosa* Douglas ex C. Lawson) and Coulter pine (*P. coulteri* D. Don) [3,4]. In Mexico, *D. brevicomis* apparently does not produce outbreaks, its populations are far less abundant, and the insect colonizes a wider range of pine species, including *P. arizonica* Engelm, *P. ayacahuite* Ehrenb. Ex Schltdl, *P. durangensis* Martínez*, P. engelmannii* Carr., *P. leiophylla* Schiede Ex Schltdl. & Cham., and *P. teocote* Schiede Ex Schltdl. & Cham. [2,5]. *Dendroctonus brevicomis* is a member of the *Dendroctonus frontalis* species complex, which is composed of *D. adjunctus* Blandford, *D. approximatus* Dietz, *D. frontalis* Zimmermann, *D. mesoamericanus* Armendáriz-Toledano and Sullivan, *D. mexicanus* Hopkins, and *D. vitei* Wood [6,7]. This bark beetle group is considered taxonomically difficult because these species have substantial morphological and biological similarities, wide intraspecific morphological variation, and distributions that overlap in Arizona and parts of Mexico and Central America [5,8,9,10].

*Dendroctonus brevicomis* was described from California specimens [11], and subsequently, an allopatric taxon morphologically similar to *D. brevicomis* was described and named *Dendroctonus barberi* [12]. These species were differentiated mainly by external morphological attributes; in particular, the presence of coarse rugosities on the elytral interspaces and impressed striae on the elytral declivity in *D. barberi*, contrasted with fine rugosities in the elytral interspaces and slightly impressed striae on the elytral declivity in *D. brevicomis*. The reported distribution of *D. barberi* included Utah, Colorado, Arizona New Mexico, and Texas [12] (p. 87 range map additionally indicated southern California and Chihuahua), whereas *D. brevicomis* was reported to occur in British Columbia, Washington, Montana, Idaho, Oregon, and California, [12,13]. However, since the differences in elytral sculpturing were not consistent either for the type specimens or for other specimens collected in the hypothesized ranges of either species (including Mexico), *D. barberi* was placed in synonymy with *D. brevicomis* [14].

Currently, *D. brevicomis* is considered a full taxon [15]; however, molecular, cross-breeding, pheromonal, morphometric, and range distribution evidence suggest that this taxon could contain unnamed cryptic species [2,6,16,17,18,19]. To build on this previous research, we performed a comprehensive analysis of intraspecific morphological variation of *D. brevicomis* across its entire distribution to test the hypothesis of extant cryptic species. We used traditional morphometric methods to analyze the variation of discrete and continuous morphological characters, as well as geometric morphometrics of three structures: Antenna, spermatheca, and seminal rod. Variation in these structures has been useful for clarifying and defining the taxonomic status of other members of this species complex.

## 2. Materials and Methods

### 2.1. Samples

**Figure 1:**
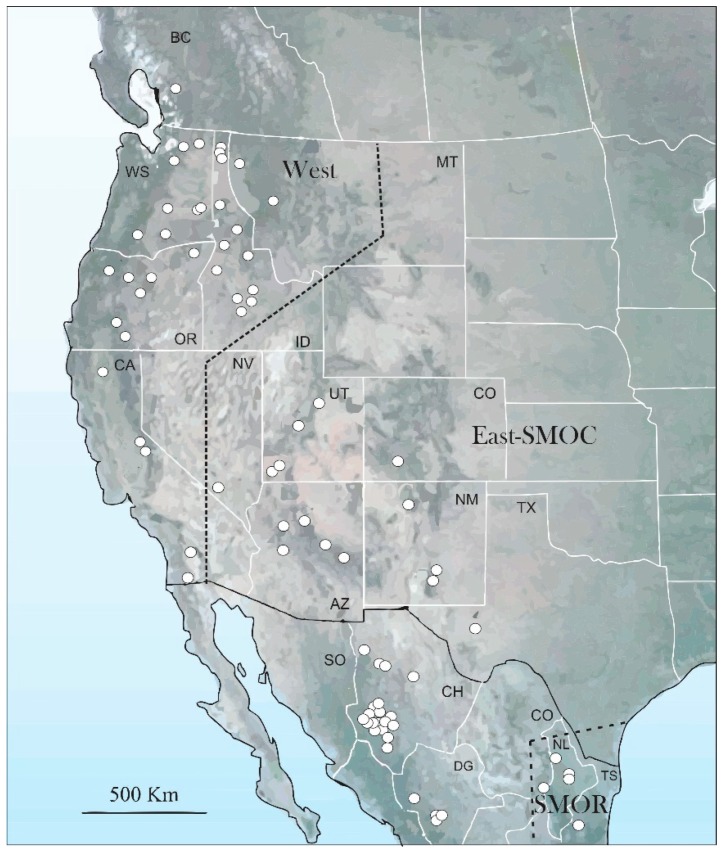
Distribution map, and populations analyzed across of distribution range of *D*. *brevicomis*.

A total of 706 adults of *D*. *brevicomis* from 81 localities in Canada, United States, and Mexico were analyzed (Figure 1, Table S1). The specimens were collected from naturally-infested trees or were donated or loaned by the following institutions: Universidad Autónoma de Chapingo (UACH), Mexico; Comisión Nacional Forestal (CONAFOR) in Jalisco, Durango and Chihuahua states, Mexico; Instituto Nacional de Investigaciones Forestales Agrícolas y Pecuarias (INIFAP), Aguascalientes, Mexico; Laboratorio de Análisis y Referencia en Sanidad Forestal (LARSF), Secretaría del Medio Ambiente y Recursos Naturales, Ciudad de Mexico, Mexico; Colorado State University, Fort Collins, Colorado, USA; C. P. Guillete Museum of Arthropod Diversity, University of Colorado, Boulder, Colorado, USA; Entomology collection of University of Colorado Museum of Natural History, Boulder, Colorado, USA; United States Forest Service, Rocky Mountain Research Station, Fort Collins, Colorado, and Southern Research Station, Pineville, Louisiana; United States Forest Service, Forest Health Protection, Boise, Idaho and Missoula, Montana; William F. Barr Entomological Museum, University of Idaho, Boise, Idaho, USA; and M. T. James Museum, University of Washington, Pullman, Washington, USA; Ministry of Forests, Lands and Natural Resource Operations, Kamloops, British Columbia, Canada. In addition, type specimens of both *D*. *brevicomis* LeConte and *D*. *barberi* Hopkins deposited at the Museum of Comparative Zoology, Harvard University and the Smithsonian National Museum of Natural History, Washington D.C. were examined.

Our initial identification of specimens as *D*. *brevicomis* was based on the taxonomic keys of Lanier et al. [6] and Armendáriz-Toledano and Zúñiga [20]. Males and females were separated by the presence of frontal tubercles on males and sexual dimorphism of the seventh abdominal tergite [21].

**Figure 2:**
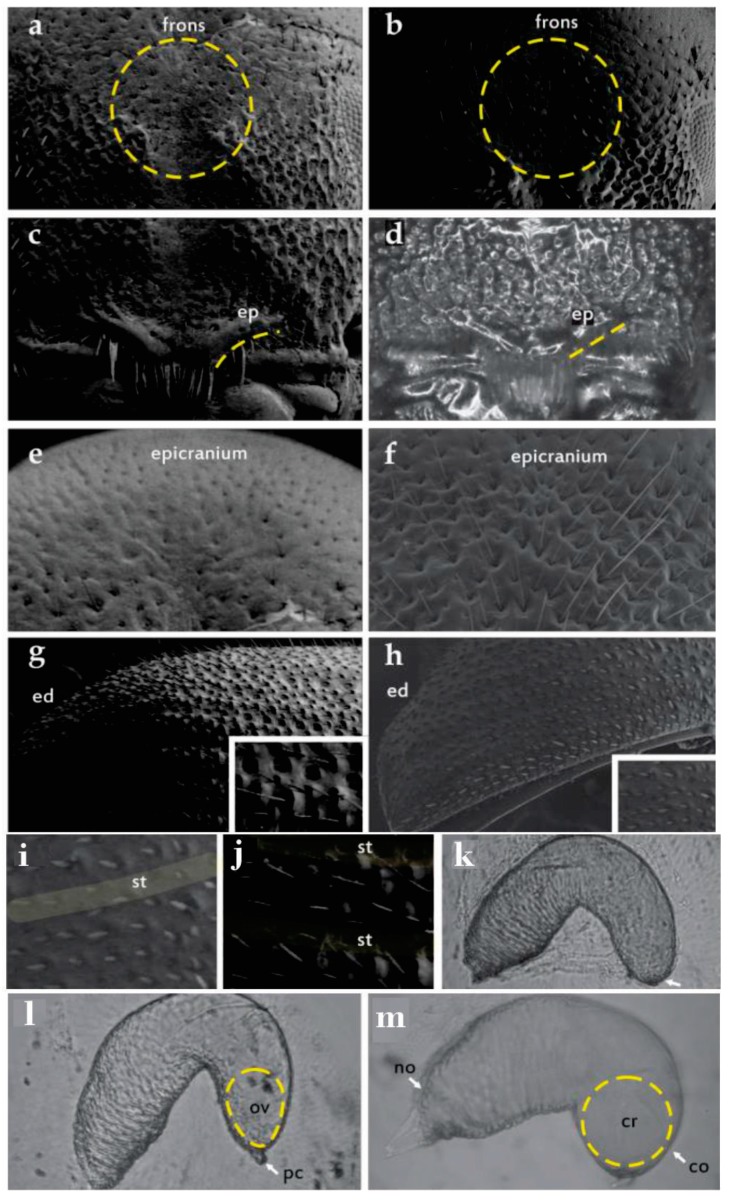
Qualitative characters evaluated in *D*. *brevicomis* populations: **FS**—Frontal sculpture (**a**,**b**); **DE**—Degree of elevation in the epistomal process, ep (**c**,**d**); **ES**—Epicranium surface (**e**,**f**); **SPD**—Size of the pubescences in the elytral declivity, (**g**,**h**); **RSP**—Thickness of the pubescences of the striae in relation to the pubescences of the interestriae (**g**,**h**); **SED**—Striae on the elytral declivity, st (**i**,**j**); **PNCS**—Proportion of the nodulus covered by striae (**k,l,m**), **CS**—Cornu shape (**l,m**): ov = oval, cr = circular; **PC**—Protuberance of cornu (**l**), no = nodulus, co = cornum.

**Figure 3:**
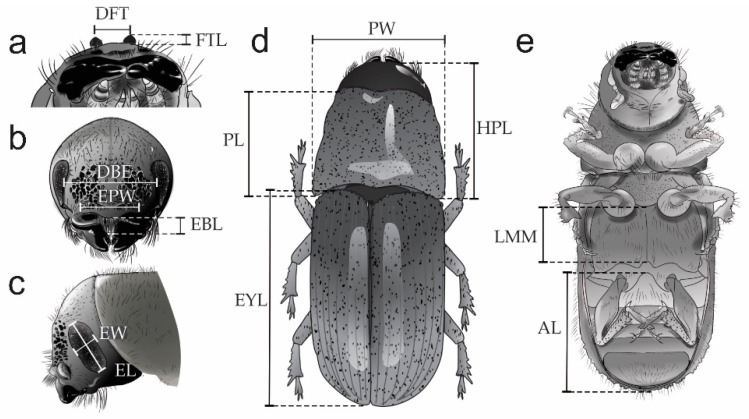
Quantitative characters evaluated in *D*. *brevicomis* populations: **FTL**—Frontal tubercles length, **DFT**—Distance between frontal tubercles, **EBL**—Epistomal brush length, **EPW**—Epistomal process width, **EW**—Eye width, **EL**—Eye length, **DBE**—Distance between eyes, **HPL**—Head-pronotum length, **PL**—Pronotum length, **PW** —Pronotum width, **LMM**—Length of the midline of the metathorax, **AL**—Abdominal length, and **EYL**—Elytra length.

Based on a comprehensive review of all specimens, 52 characters (data not shown) were selected and evaluated for potential diagnostic value; these included characters previously reported as differentiating *D*. *brevicomis* and *D*. *barberi* [12,13,14]. Most of these characters were those traditionally employed in taxonomy of Scolytinae. However, 22 of them were selected for intensive study due to their consistency and degree of variation in *D. brevicomis*: Six qualitative (1–6, described below) and 11 quantitative (12–22) characters were common to both sexes, three qualitative characters were exclusive to females (7–9), and two quantitative characters (10–11) were exclusive to males (Figure 2 and Figure 3). Qualitative characters were coded as double state for statistics analyses.

### 2.2. Characters

**1. *Frons sculpturing (FS)*** (scarce vs. abundant). Males of some species of *Dendroctonus* have numerous and prominent granulate tubercles on the lateral areas of the frons [15]. This sculpturing consists of granules, punctures, and fused granules resembling small crenulations (Figure 2a,b). Some authors have used this attribute to discriminate reliably between some members of *D*. *frontalis* complex, such as *D*. *vitei* and *D*. *mexicanus* [6,15,22].

**2. *Degree of elevation of the epistomal process (DE)*** (elevated vs. not elevated). The epistomal process is a structure immediately above the oral cavity that comprises a pair of lateral elevations or “arms” and an interposing ridge (Figure 2c,d). Process width, elevation, and inclination display important differences that are useful for distinguishing species or species groups in *Dendroctonus* [12,14,15].

**3. *Epicranial Surface (ES)*** (smooth vs. rough). The sculpture of the epicranial surface has not been evaluated previously in the genus *Dendroctonus* (Figure 2e,f).

**4. *Length of pubescence on the elytral declivity (SPD)*** (variable vs. uniform). Length of elytral pubescence has been a useful character for separating *D*. *brevicomis* populations (Figure 2g,h) [2,12,13]. Specimens from British Columbia and California were found to possess pubescence on the elytral declivity that was variable in length but did not exceed the width of interestriae, whereas specimens from Arizona, Chihuahua, Durango, and Nuevo Leon displayed a more uniform pubescence length [2].

**5. *Thickness of the pubescence of the striae in relation to the pubescence of the interestriae (RSP)*** (thin vs. thick). The surface of the elytra is covered by abundant setae of different thicknesses. In *Dendroctonus*, the setae on the elytral interspaces usually differ in thickness from those in the interstriae. This character has not been evaluated previously (Figure 2g,h).

**6. *Striae on the elytral declivity (SED)*** (impressed vs. poorly or not impressed). The degree of impression of the striae on the elytral declivity has been used to distinguish some members in the genus *Dendroctonus* [12,14,15]. Hopkins [12] proposed this as useful character for separating *D*. *barberi* and *D*. *brevicomis* (Figure 2i,j).

**7. *Proportion of the nodulus covered by striae (PNCS)*** (50% vs. >50%). In the female spermatheca, the pattern of striations can cover different proportions of the nodulus (Figure 2k,l,m). This character has been shown to differ among species of the *D*. *frontalis* complex [7,20].

**8. *Cornu shape (CS)*** (oval vs. rounded). The female spermatheca is divided into a nodulus and cornu (Figure 2l,m). The cornu is the distal portion of the spermatheca beyond the middle constriction, whereas the nodulus is the proximal portion [7,20]. Variation in cornu shape has shown to be a useful taxonomic character for separating some species within the *D*. *frontalis* complex.

**9. *Protuberance of cornu (PC)*** (absent vs. present). The proximal region of female spermathecae (nodulus) may possess a protuberance (Figure 2l). This is the first time that this character is described and shown to differentiate among *D*. *frontalis* complex members.

**10. *Length of frontal tubercles (FTL).*** This attribute was measured from the center of the median groove to the apex of the frontal tubercles (Figure 3a), in dorsal view.

**11. *Distance between frontal tubercles (DFT).*** This attribute was measured between the apices of the right and left frontal tubercles (Figure 3a), in dorsal view.

**12. *Epistomal brush length (EBL)***. This attribute was measured between the anterior and posterior edges of the epistomal brush (Figure 3b), in frontal view.

**13. *Epistomal process width (EPW)***. This attribute was measured from the left and right lateral margins in the epistomal process (Figure 3b), in frontal view.

**14. *Distance between the eyes (DBE).*** This attribute was measured between the internal margins of the central region of the eyes (Figure 3b), in frontal view.

**15. *Eye width (EW)***. This attribute was measured between the lateral margins of the central region of the right eye (Figure 3c), in lateral view.

**16. *Eye Length (EL).*** This attribute was measured between the dorsal and ventral margins of the right eye (Figure 3c), in lateral view.

**17. *Head-pronotum length (HPL).*** In the case of females, this attribute was measured in dorsal view from the frons (including frontal tubercles in males) to the posterior, lateral margin of the pronotum. The measurement could be slightly influenced by the angle that the head happens to be in, and therefore it must be measured based on the images of Figure 3d.

**18*. Pronotum length (PL).*** This attribute was measured along the median line of the pronotum from the anterior to the posterior margin (Figure 3d), in dorsal view.

**19*. Pronotum width (PW)***. This attribute was measured at the widest portion of the pronotum in dorsal view, from left to right margins, (Figure 3d).

**20*. Elytra length (EYL)***. This attribute was measured from the anterior margin of the elytra to the posterior terminus of the elytral declivity (Figure 3d), in dorsal view.

**21. *Abdominal length (AL)***. This attribute was measured between the intercoxal process and the posterior tip of the venter (Figure 3e), in ventral view.

**22. *Length of the midline of the metathorax (LMM).*** This attribute was measured from the sternellar area to sternellar piece in the metathorax (Figure 3e), in ventral view.

Characters were observed and measured at 100–400× with phase contrast microscopy (Carl Zeiss, Oberkochen, Germany), and photographed with a Coolpix 5000 camera (Nikon, Tokyo, Japan). Antenna, elytra, and genitalia were removed and mounted following the protocols previously reported for these structures in other species [7,23]. The elements of elytral sculpture (punctures, granules, pubescence, etc.) were measured and quantified directly in slides with an ocular micrometer under a phase contrast microscope (400×). Details of characters were photographed with an environmental scanning electron microscope (ESM Evo**^®^** 40VP; Zeiss, Ontario, CA, USA).

### 2.3. Morphometrics Analysis

To characterize the main geographical trends of morphological variation among specimens, we performed three principal coordinates analyses (PCoA): Females and males together (*n* = 706), and females (*n* = 340) and males (*n* = 366) alone (Table S1). These analyses used pairwise Gower’s distance [24] estimated from corresponding qualitative and quantitative characters (17 for sexes combined, 17 for females, and 19 for males). Quantitative characters were log transformed because they did not meet the criteria of normality. Additionally, we used a canonical variate analyses (CVAs) to determine to what extent these attributes explained the possible geographical regionalization of specimens. For this analysis, we calculated average values and variances for each character clustered by the putative geographic groups defined with PCoA. Lastly, we looked for statistical differences among geographical groups with an analysis of similarities (ANOSIM) and pairwise Hotelling’s T non-parametric tests [25].

Lastly, to assess the relative taxonomic weight of qualitative and quantitative characters employed, we examined statistical differences with chi-square contingency tables and ANOVA tests, respectively [25]. As with PCoA, we analyzed males and females together and separately within the putative geographical groups.

### 2.4. Geometric Morphometrics

We evaluated whether shape variation of the antennal club (*n* = 325), seminal rod (*n* = 202 dorsal view, 247 lateral view), and spermatheca (*n* = 203) (Table S1) segregated specimens into geographical groups. Structures were oriented in the same direction for photographs (Figure S1). Few homologous points were available in these features for landmarks (lds), thus we also used semilandmarks (smlds), which are points assigned along a geometric curve, edge, or surface of a feature [26,27]. Smlds were defined by superimposing radial lines (“fans”) or parallel lines (“combs”) onto digitized photographs of the antennal club, seminal rod, and spermatheca using MakeFan6 of the Integrated Morphometrics Package (IMP) [28].

A total of eight lds (2–3; 5, 10; 11, 16; 17, 22) and 16 smlds (1–4; 7–9; 12–15; 18–21; 23–24) were used to characterize the antennal club configuration (Figure S2a). To define smlds, we traced a straight line between lds 11 and 16 located at the intersection of the extremes of the second sensorial band and outline of this structure, then by applying a comb overlay, we projected from it six equidistant, perpendicular lines. Smlds were located at every intersection between these lines and sensory bands, as well as the outline of antennal club (Figure S2a).

For the spermatheca configuration we used two lds (1, 14) and 31 smlds (2–13, 15–33) (Figure S2b). Smlds were defined by drawing a straight line between landmarks 1 and 14 (the apices [curvature maxima] of the nodulus and cornu, respectively), and from the midpoint of this line, projecting a fan with 21 radiating lines at equal angles (Figure S2b).

For seminal rod configuration in dorsal view, we used 10 lds (1–2, 8, 13, 21, 29, 34, 40–42) and 32 smlds (3–7, 9–12, 14–20, 22–28, 30–33, 35–39) (Figure S2c). Three straight lines were drawn between well-defined lds to define smlds. The first line was between the apex of the right arm of the seminal valve and the intersection of the outline of the seminal rod body and right arm (lds 8 and 13, respectively); the second line, between the apex of the seminal rod body and the maximum curvature of the seminal valve (lds 21 and 41, respectively); the third line was the left-side equivalent of the first line (lds 29 and 34). From these lines, we projected 6, 10, and 6 perpendicular lines, respectively.

Lastly, for seminal rod configuration in lateral view, we used 5 lds (1, 11, 19, 23, 27) and 20 smlds (2–10, 12–18, 20–22, 25–26). Smlds were defined as the intersections of the structure outline with 26 equidistant lines drawn perpendicular to a line between the proximal and distal ends of the structure (lds 1 and 11, respectively) (Figure S2d).

For each specimen, the x, y coordinates of lds and smlds from each morphological structure were captured using the software TpsDig ver.140 [29]. To minimize the tangential variation of smlds, a coordinate adjustment was performed in SemiLand 6 [28]. Procrustes superimposition was performed in CoordGen6 to remove the effect of size, position, and rotation on position of coordinates of the individual configurations [26,27].

To obtain new variables that quantified the highest percentage of shape variation of these structures, relative warps analysis (RWA) was performed in PAST 3.12 [30] using the adjusted x, y coordinates matrix for both specimens and localities [27]. RWAs were performed using paired variance–covariance matrices among specimens or localities. Shape variation was plotted using the first two relative warps (RW1 vs. RW2; data not shown), and the change in the structure’s configuration was visualized by thin-plate spline deformation grids in PAST 3.12.

Canonical variate analyses (CVAs) were performed to test whether the shape variation of these structures could be used to discriminate among possible geographic groups. Based on Procrustes results of the antennal club, seminal rod, and spermathecae, the specimens of different localities analyzed were a priori assigned to possible geographic groups. CVAs of specimens were performed from the matrix x, y configurations. Non-parametric multivariate analyses of variance (PERMANOVA) and post-hoc pairwise Hotelling’s T-tests [27] were performed to evaluate differences among the groups recovered by CVAs. All multivariate analyses were performed using PAST 3.12.

## 3. Results

### 3.1. Multivariate Analysis of Non-Geometric Morphological Data

**Figure 4:**
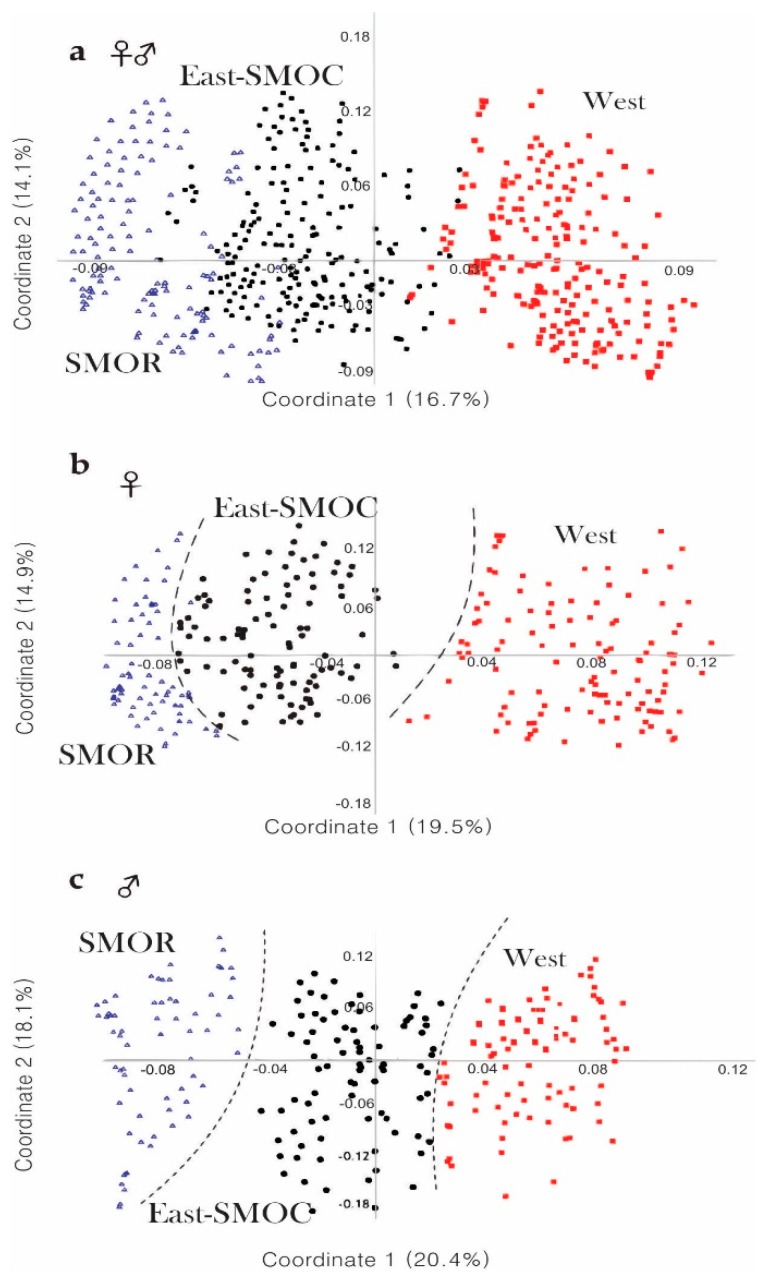
Scatters plots from principal coordinate analysis (PCoA) from qualitative and quantitative characters in *D*. *brevicomis* populations. (**a**) Among specimens of both sexes, (**b**) among females, and (**c**) among males.

The first three coordinates of the PCoA considering the 17 characters common to both sexes explained 43.8% of total variation (PCo1, 16.7%; PCo2, 14.1%; PCo3, 13%). The scatter plot of the two first components (PCo1 vs. PCo2) showed partial separation of the specimens into three recognizable geographical groups (Figure 4a). The first included specimens of the populations located in the western coastal states of the USA and British Columbia including Idaho, Montana, and southern Nevada (hereafter, “West group”); the second included specimens of the states adjacent to those of the west coast (Utah, Colorado, Arizona, New Mexico, and Texas), as well as those from Chihuahua and Durango states in the Sierra Madre Occidental (SMOC) in the North of Mexico (East-SMOC group); the third included specimens from Nuevo Leon, Coahuila, and Tamaulipas states in the Sierra Madre Oriental (SMOR) in northern Mexico (SMOR group). The specimens of both West and SMOR groups presented a slight overlap with those of the East-SMOC group (Figure 4a). Significant differences were found among these geographical groups (ANOSIM, *R* = 0.501; *p* ≤ 0.001). Pairwise Hotelling’s T-tests support differences between groups: West vs. East-SMOC groups (*R* = 0.440, *p* ≤ 0.001), West vs. SMOR (*R* = 0.706, *p* ≤ 0.001), and East-SMOC vs. SMOR (*R* = 0.336, *p* ≤ 0.001).

In the case of PCoA for females, the first three principal coordinates explained 44.9% of total variation (PCo1, 19.5%; PCo2, 14.9%; PCo3, 10.4%). The scatter plot of the first two coordinates (PCo1 vs. PCo2) partially recovered the same groups found in the analysis of sexes combined (Figure 4b), but with a slight overlapping between East-SMOC and SMOR groups. The West group formed a cluster distinct from the other groups. Significant differences were found among these groups (ANOSIM, *R* = 0.438, *p* ≤ 0.001). Pairwise Hotelling’s T-tests supported these differences: West vs. East-SMOC (*R* = 0.486, *p* ≤ 0.001), West vs. SMOR (*R* = 0.666, *p* ≤ 0.001), and East-SMOC vs. SMOR (*R* = 0.26, *p* ≤ 0.001).

In the case of male PCoA, the first three coordinates explained 48.5% of total variation (PC1, 20.4%; PC2, 18.1%; PC3, 10%). The scatter plot of the two first coordinates (PCo1 vs. PCo2) indicated that SMOR formed a cluster distinct from East-SMOC and West, but that the latter two groups overlapped (Figure 4c). Significant differences were found among geographical groups (ANOSIM, *R* = 0.588, *p* ≤ 0.001). Pairwise Hotelling’s T-tests supported these differences: West vs. East-SMOC (*R* = 0.412, *p* ≤ 0.001), West vs. SMOR (*R* = 0.772, *p* ≤ 0.001), and East-SMOC vs. SMOR (*R* = 0.551, *p* ≤ 0.001).

### 3.2. Relative Taxonomic Weight of Individual Characters

The qualitative characters FS, SPD, RSP, SED, and PSS differed significantly (*p* < 0.05) among specimens from different geographical groups; however, only SPD was an exclusive character of the West group (Table S2). Beetles from this group possessed 2–3 sizes of pubescence (SPD) on the elytral declivity, whereas specimens from the other groups possessed only one (Figure 2g,h).

The ANOVA and Tukey tests showed that ten quantitative characters common to females and males (EBL, EPW, EL, DBE, HPL, PL, PW, EYL, AL, LMM) displayed statistically significant differences in at least one geographical group; however, these were not always presented in the same groups (Table S3a). The individuals from SMOR showed higher average values in these characters than the specimens of other geographical groups. Specimens from East-SMOC group had lower average values of epistomal brush length (EBL), epistomal process width (EPW), eye length (EL), and pronotum width (PW) than individuals of West and SMOR groups; and they were smaller than individuals of these groups, except in the EYL and LMM characters. Specimens of the West group typically displayed intermediate average values between East-SMOC and SMOR groups.

Seven (EL, HPL, PL, PW, EYL, AL, MML) of 11 characters were statistically different among females (Table S3b). The differences were also concentrated in measures of size, with SMOR females being larger than those from West and EAST-SMOC groups. The females from West and East-SMOC were very similar, showing significant differences in only four characters (EL, HPL, PW, and AL). In the case of males, seven (DFT, EBL, EPW, EL, PW, AL, MML) of 13 characters were statistically different (Table S3c); however, only three of them were different (PW, AL, MML). SMOR males were slightly larger than those of males from West and East-SMOC groups, and similar to females, the West males displayed intermediate mean values between East-SMOC and SMOR groups.

### 3.3. Geometric Morphometrics

**Figure 5:**
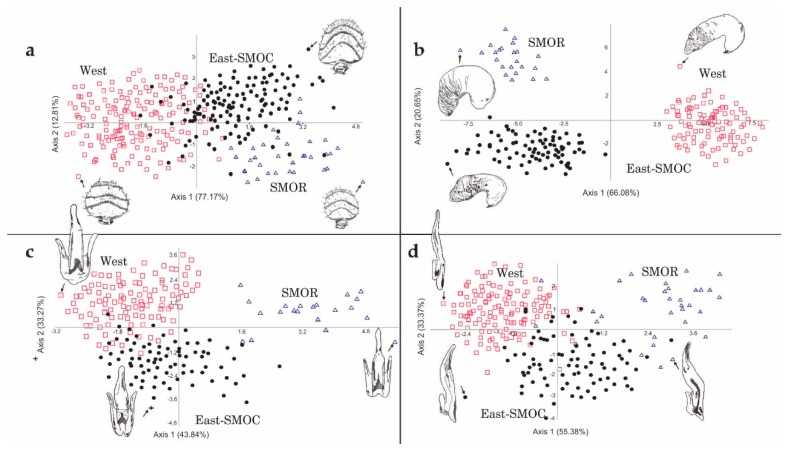
Scatter plots of first and second relative warps in the principal components analysis among *D. brevicomis* specimens: (**a**) Females–males antennae, (**b**) spermathecae, and (**c**,**d**) seminal rod in lateral and dorsal view.

The first four of the relative warp analysis (Figure 5 and Figure S2) explained 58.7% of the observed total variation in atn (RW1, 23%; RW2, 17.1%; RW3, 9.6%; RW4, 9%), 64.8% in srdv (RW1, 28.5%; RW2, 17.8%; RW3, 11.5%; RW4, 7%), 65.7% in srlv (RW1, 28.5%; RW2, 15.4%; RW3, 11.9%; RW4, 9.9%), and 74.6% in spmt (RW1, 29.3%; RW2, 24.2%; RW3, 13%; RW4, 8.1%).

**Figure 6:**
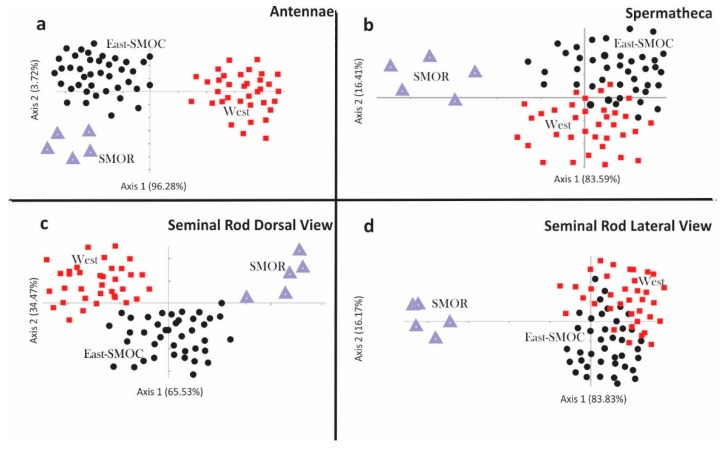
Scatter plots of individuals of different *D*. *brevicomis* populations in the first two canonical axis: (**a**) Females–males antennae, (**b**) spermatheca, and (**c**,**d**) seminal rod in dorsal and lateral view, respectively.

The configurations for antennae (atn), seminal rod in dorsal view (srdv), seminal rod in lateral view (srlv), and spermathecae (spmt) for *D*. *brevicomis* showed several anatomical regions where the shape variation was concentrated. RW1 of the deformation grid of atn corresponded to changes in the degree of curvature of the sensory bands in the antennal club and in the distance between the second and third sensory bands (Figure 5a and Figure S2a). RW2 corresponded to deformations in the proximal margin of the antennal club, the distance between the proximal margin of the antennal club and the first sensory band, and deformations of the lateral margins of the antennal club. The bivariate scatter plot of the RWs of atn, recovered specimens in West, East-SMOC, and SMOR as three partially overlapping clusters (Figure 5a and Figure S2a). In the CVA, all groups had the highest correct classification of individuals (100%) (Figure 6a). PERMANOVA supported statistical differences in the shape of this structure between West vs. SMOR (*p* ≤ 0.0001), West vs. East-SMOC (*p* ≤ 0.001), and East-SMOC vs. SMOR (*p* ≤ 0.001).

RW1 of the deformation grid of spmt corresponded to deformations in the degree of curvature in the nodulus margins, nodulus length, and curvature and symmetry of the cornu (Figure 5b and Figure S2b). RW2 corresponded to variation in the length of the nodulus, the distance between nodulus and cornu, and the length and symmetry of the cornu. The plot of RWs of spmt separated all three geographical groups (Figure 5b). In the CVA, there was a slightly high percentage (80–90%) of correct classification of individuals from West and East-SMOC, but full classification of all SMOR specimens correctly classified (100%) (Figure 6b). PERMANOVA supported statistical differences in the shape of this structure between West vs. SMOR (*p* ≤ 0.0001) and East-SMOC vs. SMOR (*p* ≤ 0.002), but not between West vs. East-SMOC.

RW1 of the srdv deformation grid corresponded to changes in the length of the lateral arms of the seminal rod valve and in the thickness at the center of the seminal rod body. RW2 corresponded to changes in the length of the seminal rod valve and symmetry of the lateral arms of the seminal rod valve (Figure S2c). The plot of RWs of srdv indicated slight overlapping among all three geographical groups (Figure 5c). However, CVA indicated 100% accurate classification of specimens to groups (Figure 6c). PERMANOVA supported statistical differences in the shape of this structure among all geographic groups: West vs. East-SMOC (*p* ≤ 0.032), West vs. SMOR (*p* ≤ 0.0002), and East-SMOC vs. SMOR (*p* ≤ 0.0001).

RW1 of srlv corresponded to variation in width at the center of the seminal rod body and the seminal valve. RW2 corresponded to variation in the degree of curvature of the dorsal and ventral margins of the seminal rod body, as well as in the length and thickness of the seminal valve (Figure S2d). The plot of RWs of srlv showed some overlap among all three geographical groups (Figure 5d). The CVA analysis correctly classified 89.5% of specimens to geographic group (Figure 6d). PERMANOVA supported statistical differences in the shape of this structure between West vs. SMOR (*p* ≤ 0.0001), and East-SMOC vs. SMOR (*p* ≤ 0.0001).

## 4. Discussion

Our PCoA of quantitative and qualitative with sexes combined indicates continuous phenotypic variation in populations of *D. brevicomis*, as indicated by Wood [14]. However, when females and males were analyzed separately, we recovered three geographical groups defined by discontinuities in morphological variation corresponding to West (females distinct), SMOR (males distinct), and East-SMOC (geographic zone occupied exclusively by the remaining specimens) (Figure 4). These findings indicate that variation is, in fact, not continuous; rather, natural division into groups is dependent on the sex examined. One other study has explored the relative contribution of each sex in the quantitative morphological differentiation of *Dendroctonus* species; however, no difference was observed [7].

The evaluation of nine discrete characters indicated that only one (size of the pubescence of the elytral declivity, SPD) was useful for separating West specimens from the other groups. West specimens possessed pubescence of differing lengths in the interstriae of the elytral declivity, whereas insects from other groups had only one length. Since this feature is external and readily observed at low magnification, it represents a taxonomic character that is particularly suitable for identification in the field. Although Wood [14] reviewed few specimens from Mexico, he found no reliable ways to distinguishing *D. brevicomis* (West group) from *D. barberi* (East specimens within East-SMOC Group).

Quantitative morphological characters showed considerable geographic variation among the three geographic groups. However, quantitative characters corresponding to body size possessed significant differences in average values among groups, with SMOR specimens being on average larger than those of the other groups. Furthermore, there was greater similarity in size between West and East-SMOC specimens than between either of these groups and SMOR. Univariate and multivariate statistical analyses have frequently been used to identify morphological differences among species of scolytines including *Dendroctonus*: *D*. *monticolae* Hopkins and *D*. *ponderosae* Hopkins [31], *D. valens* LeConte and *D. terebrans* Olivier [32], *D*. *parallelocollis* and *D*. *approximatus* [33], as well as species of the *D. frontalis* complex [6,34].

### 4.1. Geometric Morphometrics

Our findings on shape variation of the antennae and seminal rod in lateral view partially confirm the presence of three geographical groups, because scatter plots of the relative warps of these structures show significant segregation of specimens within the three geographic groupings (Figure 5). Antennal club shape has received little attention as a taxonomic character in other scolytids including *Dendroctonus* species. In those studies where antennal club morphology has been used, significant differences in antennal club shape have been found between sibling species, including *D*. *valens* Le Conte and *D*. *rhizophagus* Thomas & Bright [23], *D. frontalis* Zimmermann and *D. mesoamericanus* Armendáriz-Toledano & Sullivan [7], and *D*. *approximatus* Dietz and *D*. *parallelocollis* Chapuis [33]. In addition, in *D. vitei* Wood, the presence of sensillae clustered into pit craters is a taxonomic attribute unique within the *Dendroctonus frontalis* species complex [35].

Likewise, despite substantial intraspecific variation, seminal rod shape has been used to separate members of the *D*. *frontalis* complex [2,7], confirming the taxonomic value of this structure [6,15,36,37]. In the present study, the scatter plot of seminal rod shape in dorsal view only partially recovered three geographic groups (Figure 5c,d). East-SMOC individuals exhibited broad variation in shape of the seminal rod body that overlapped with that of the West and SMOR groups. However, there were consistent differences among groups focused in the seminal rod valve (Figure 6d), and SMOR and West were well resolved in dorsal view.

Additionally, the three geographical groups could be discriminated by shape of the female genitalia (Figure 5b). Spermathecae of West females possessed a nodulus that was wider than the cornu and had an oval shape, East-SMOC females had a nodulus with rounded anterior and posterior edges and a distally-widened, ovate cornu, and SMOR females possessed a nodulus that was slightly wider than the cornu, and a cornu similar to East-SMOC specimens. The female genitalia have rarely been used in the taxonomy of the genus *Dendroctonus*; however, some discrete attributes have proven to be useful for separating *D*. *frontalis* from *D*. *mexicanus* [38], *D*. *frontalis* from *D. mesoamericanus* [7], *D. vitei* from *D. mexicanus* [39], and *D. approximatus* from *D*. *parallelocollis* [33].

### 4.2. Taxonomic Considerations

Traditionally, the species status of members of the genus *Dendroctonus* has been supported by biological and ecological data, together with external morphological characters [12,15,40]. In addition, cytogenetic evidence, crossbreeding tests, molecular and biochemical data, and comprehensive analysis of morphological variation, e.g., [6,7,39,41], have been used to confirm, segregate, or describe new species.

Previous morphological [12], biological [6], genetic [16], pheromone [17], and molecular [18] studies support the conclusion that *D. brevicomis* is constituted by two distinct taxa corresponding to our West and East-SMOC groups within the USA; however, these studies did not analyze specimens across the entire range of *D. brevicomis*. Taking into consideration the genetic data of Kelley et al. [16] and available data on distribution, Bright [42] designated populations corresponding to our West and East-SMOC groups within the USA as subspecies of *D. brevicomis* in his taxonomic monograph of the Bark and Ambrosia Beetles of the West Indies.

Our research further demonstrates that *D. brevicomis* can be morphologically differentiated into a third geographically-delimited group (SMOR) in northeastern Mexico that appears to be distinct from the groups whose ranges are limited to or include the USA (i.e., West and East-SMOC, respectively). The specimens from these geographic areas displayed non-overlapping quantitative characteristics of external and reproductive morphology. However, we discovered only one qualitative character (pubescence length of the elytral declivity) that allowed reliable separation of the West group from the two other groups, and we found no such character to reliably separate East-SMOC from SMOR.

These morphological differences between specimens from West and East-SMOR/SMOC support the hypothesis that *D. brevicomis* is integrated by at least two different species existing on either side of the Great Basin, Mojave, and Sonoran deserts: *D. brevicomis* and *D. barberi,* as originally designated by Hopkins [12] and then synonymized by Wood [14]. Several lines of evidence support this hypothesis. First, genetic differences between populations on either side of the three western American deserts [16,18] are similar in degree to those existing between recognized sibling species or subspecies of *Dendroctonus* or other bark beetles [43,44,45,46]. Second, the composition of the aggregation pheromone differs between these populations [17,19], with the major female-produced component of the aggregation pheromone of populations west of the Great Basin being *exo*-brevicomin, and that of populations east of the Great Basin being *endo*-brevicomin [17,19]. Third, our observations in the field indicate that West specimens produce subtransversely-winding parental tunnels, whereas parental tunnels of East-SMOC specimens are distinctly transversely winding and more aligned with the bole, as was reported previously for *D. brevicomis* and *D. barberi,* respectively, by Hopkins [12].

Therefore, based on morphometric analyses in this paper, our observations of gallery construction, and the earlier genetic and chemical ecology evidence [2,16,17,18,19], we removed *Dendroctonus barberi* Hopkins from synonymy with *Dendroctonus brevicomis* LeConte and updated the descriptions for both taxa. In addition, due to broad morphological similarity observed between populations of East-SMOC both in Mexico and USA, we included both in *D. barberi*, although Mexican populations of this group were not analyzed in previous genetic or chemical ecology studies. The type specimens for *D. brevicomis* (the West Group of the present study) were deposited at the Harvard University Museum of Comparative Zoology, whereas those of *D. barberi* (East-SMOC Group of the present study) were deposited at the Smithsonian National Museum of Natural History.

### 4.3. Redescriptions

#### 4.3.1. *Dendroctonus brevicomis* LeConte 1876

The redescription of this species is based on a sample of 133 females and 133 males. Photos and illustrations were taken and drawn directly from these specimens, and the external morphology cross-checked with type specimens.

#### 4.3.2. Description of Female

**Figure 7:**
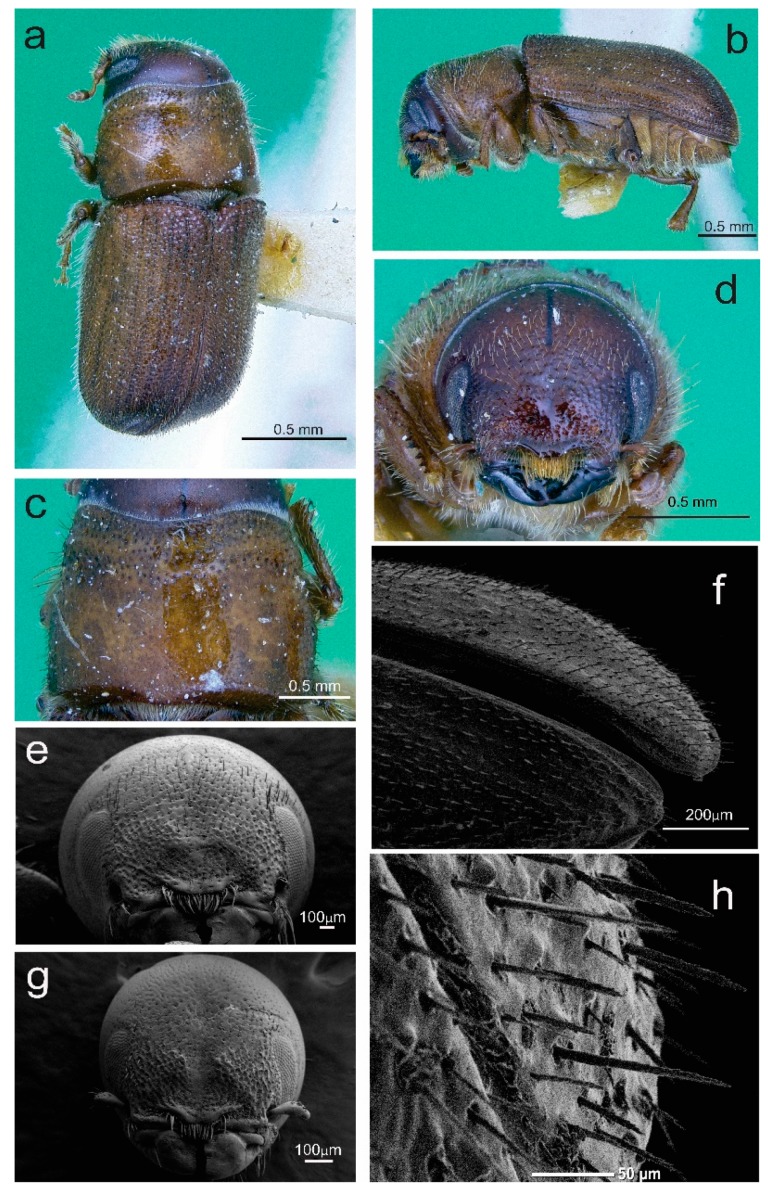
*Dendroctonus brevicomis*: (**a**) Female in dorsal view, (**b**) female in lateral view, (**c**) female pronotum, (**d**) frons sculpture of female, (**e**) female frons, (**f**) elytral declivity, (**g**) male frons, (**h**) elytra in dorsal-lateral view. Photos **a**–**d** (only existing paratype of *D. brevicomis*).

**Figure 8:**
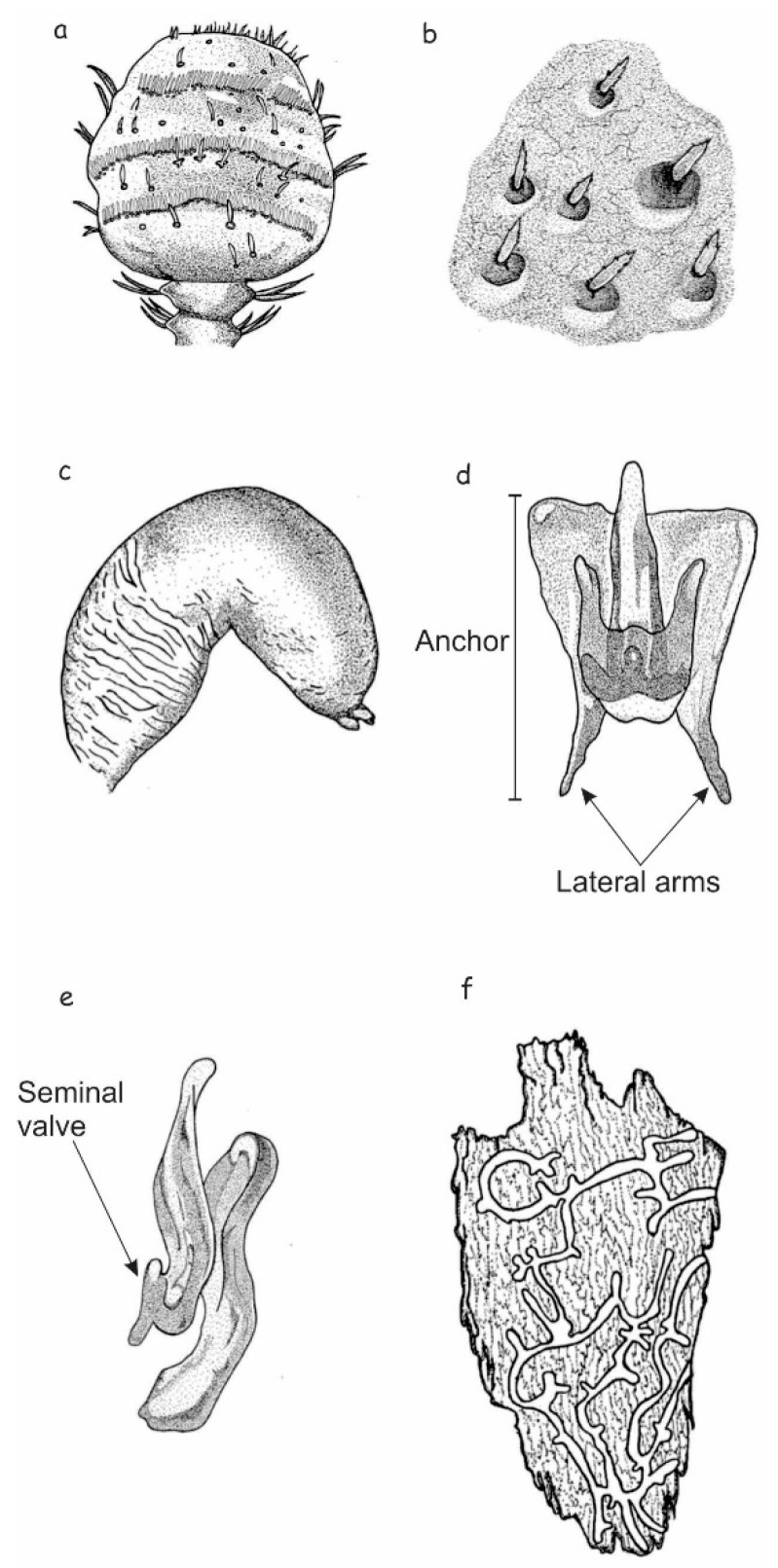
*Dendroctonus brevicomis*: (**a**) Antennal club, (**b**) elytral sculpturing and setae, (**c**) spermatheca, (**d**,**e**) seminal rod in dorsal and lateral view, (**f**) parental tunnels in host bark.

Total length, 2.04–5.08 mm ($\bar{X}$ = 3.94 mm); 2.5 times longer than wide; head black to brown; pronotum lighter in color than head, medium brown; elytra with same color as pronotum (Figure 7a–c); chromosome formula = 5AA + XX. Head: Length, 0.23–0.40 mm ($\bar{X}$ = 0.31 mm); concave frontal region that extends above the epistomal process to the upper level of the eyes separated by a deep median groove; surface of epicranium and vertex covered with small punctures, deep and variable in diameter (Figure 7d,e); vertex and frons completely convex in lateral view. Width of epistomal process, 0.18–0.60 mm ($\bar{X}$ = 0.36 mm), 0.5 times distance between eyes; arms of epistomal process slightly elevated and oblique, ~24° from horizontal (Figure 7d,e); frons surface rough, shiny, or dull, with few punctures of variable size; dense brush of yellow setae present on underside the epistomal process; head with yellow pubescence of variable size, sparsely distributed around frons; pubescence less abundant on area immediately above epistomal process (Figure 7d,e); largest setae distributed on vertex, those on lateral areas around eyes and surrounding the epistomal process small or intermediate in size. Antenna: Composed of scape, funicle, and club; funicle articulate with five antennomeres; club with four fused antennomeres, these last divided by three curved bands of sensillae (Figure 8a). Pronotum: Length, 0.56–1.58 mm ($\bar{X}$ = 1.05 mm), width 0.83–1.88 mm ($\bar{X}$ = 1.53 mm), the anterior region scarcely constricted and widest on posterior third; the dorsal surface with sparse, deep punctures (Figure 7c); on lateral median areas, punctures less defined with smooth, shiny areas present, lateral punctures on preepisternal and episternal areas shallower, less abundant, and reduced in diameter (Figure 7b); the anterolateral region of pronotum (preepisternal area) smooth; a transverse, elevated callus present along anterior margin, bulging slightly both laterally and dorsally; dorsal anterolateral zones of pronotum convex. Elytra: Length, 1.25–3.10 mm ($\bar{X}$ = 2.47 mm), ~1.6 times longer than wide, 2.2 times longer than pronotum; sides straight on basal two-thirds, broadly rounded behind; declivity convex with striae weakly to strongly impressed (Figure 7f); punctures with diameter of striae I 19–21 μm ($\bar{X}$ = 20 μm), surface between smooth punctures, punctures spaced 28–33 μm ($\bar{X}$ = 30 μm) apart; surface of interstriae with weakly or strongly marked, rugged, small punctures and crenulations. Punctures on declivital interstriae well defined and smaller than those of striae (Figure 7f–h); crenulations in interstriae of variable size, more defined and abundant in anterior area of elytral disc and scarce or absent towards posterior of elytral disc (Figure 7h and Figure 8b); setae in interstriae I–III of elytral declivity with more than one size class (typically 2–3), although generally short and color yellow to amber in mounted specimens (Figure 7f); setae on elytral disc and declivity with sawed edge (Figure 8e). Length of the setae usually greater than twice distance between them. Diameter of the punctures in the striae II on the elytral declivity 0.018–0.020 ($\bar{X}$ = 0.019 μm), distance between punctures in the striae II 0.027–0.032 ($\bar{X}$ = 0.03 μm), number of pubescences in the striae II 14–34 ($\bar{X}$ = 26), length of pubescences in the elytral declivity 0.029–0.041 ($\bar{X}$ = 0.036 μm), width of pubescences in the elytral declivity 0.0052–0.0057 ($\bar{X}$ = 0.005 μm), width of the intestria II in the distal 0.07–0.107 ($\bar{X}$ = 0.091 μm), an proximal region 0.037–0.092 ($\bar{X}$ = 0.07 μm). Genitalia: Spermatheca reniform, divided into proximal nodulus and distal cornu, nodulus wider than cornu; surface of nodulus transversely covered by striations, not aggregate and covering almost entire nodulus; surface of cornu with few striations; cornu oval in lateral view (Figure 8c).

#### 4.3.3. Description of Male.

Total length, 2.5–5.05 mm ($\bar{X}$ = 3.79), 2.5 times longer than wide; body color similar to the female; chromosome formula = 5AA + neo-XY. Head: Similar to female, except slightly greater in length, 0.24–0.84 mm ($\bar{X}$ = 0.43). Frons with median groove and elevations with apical tubercles on either side; frontal tubercles evident, one on each side of the middle furrow, some of them fused; width of epistomal process 0.22–0.55 mm ($\bar{X}$ = 0.38 mm), slightly wider than in females, 0.44 times the distance between eyes (0.54–0.97 mm, $\bar{X}$ = 0.83 mm); arms of epistomal process slightly elevated or almost flat (Figure 7g). Distance between eyes 0.54–0.97 mm ($\bar{X}$ = 0.83 mm), eyes width 0.12–0.51 mm ($\bar{X}$ = 0.22 mm), eyes length 0.22–0.61 mm ($\bar{X}$ = 0.50 mm). Antenna: Similar to the female (Figure 8a). Pronotum: Similar to female, except length 0.65–1.24 mm ($\bar{X}$ = 0.99), width from 1.08–1.88 mm ($\bar{X}$ = 1.48); transverse callus weak or unapparent. Elytra. Similar to the female, except in the length from 1.63–2.97 mm ($\bar{X}$ = 2.36), 1.6 times longer than wide, 2.3 times longer than pronotum; sides parallel on basal two-thirds, rather broadly rounded behind; declivity convex with striae weakly impressed; vestiture and sculpture of elytral declivity similar to females. Genitalia: Seminal rod composed of four sclerotized structures: Tegmen, spicule, penis, and accessory apparatus (seminal rod and seminal rod anchor). The general anatomy of tegmen, spicule, and penis similar to that describe for *Dendroctonus ponderosae* Hopkins [47]. Sclerotized plate (seminal rod anchor) positioned dorsally of the seminal rod (Figure 8d). This structure displays two curved lateral arms in dorsal view, which are joined to the seminal rod base. Dorsal process of the seminal rod in dorsal view (seminal rod body) enclosed within this triangular plate (Figure 8d). Seminal rod entire and slightly shorter in seminal rod body than in *D*. *barberi*, with a seminal rod body rounded toward the apex; in dorsal view, length of seminal rod body from four to six times the length of seminal valve (Figure 8d); lateral arms of seminal rod ending in a pointed and not fused. The lateral view of seminal rod body abruptly narrows distally; seminal valve of the seminal rod body with a projection (Figure 8e).

Gallery. Parental tunnels subtransversely-winding (Figure 8f) [12]. Larval galleries short, narrow, and on both sides of parental gallery.

Distribution. It occurs in the USA and Canada: From southern British Columbia along the western coastal states of the USA to southern California, as well as in the states of Idaho and Montana.

#### 4.3.4. Dendroctonus Barberi Hopkins 1909

The redescription of this species is based on a sample of 119 females and 119 males. Photos and illustrations were taken and drawn directly from these specimens and the external morphology cross-checked with type specimens.

#### 4.3.5. Description of the Female

**Figure 9:**
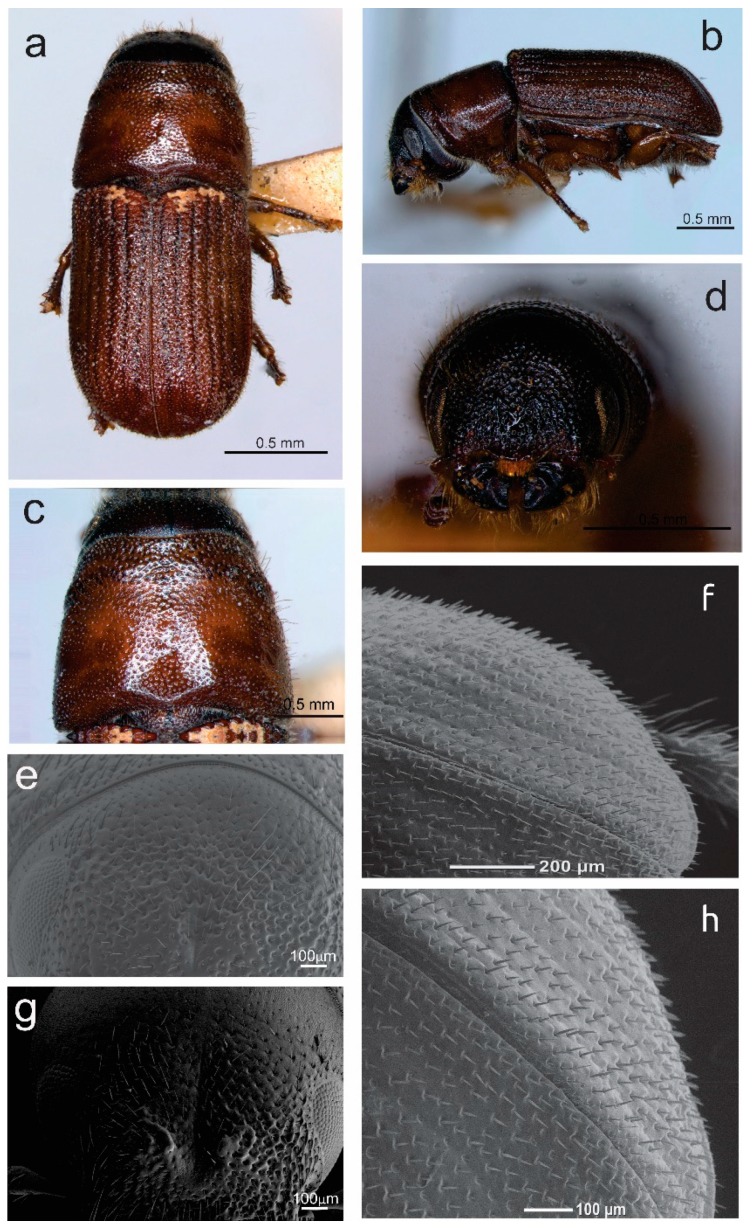
*Dendroctonus barberi*: (**a**) Female in dorsal view, (**b**) female in lateral view, (**c**) female pronotum, (**d**) frons sculpture of female, (**e**) female frons, (**f**) elytral declivity, (**g**) frons sculpture of male, (**h**) elytra in dorsal view. Photos **a**–**d** (from type specimens of *D. barberi*).

**Figure 10:**
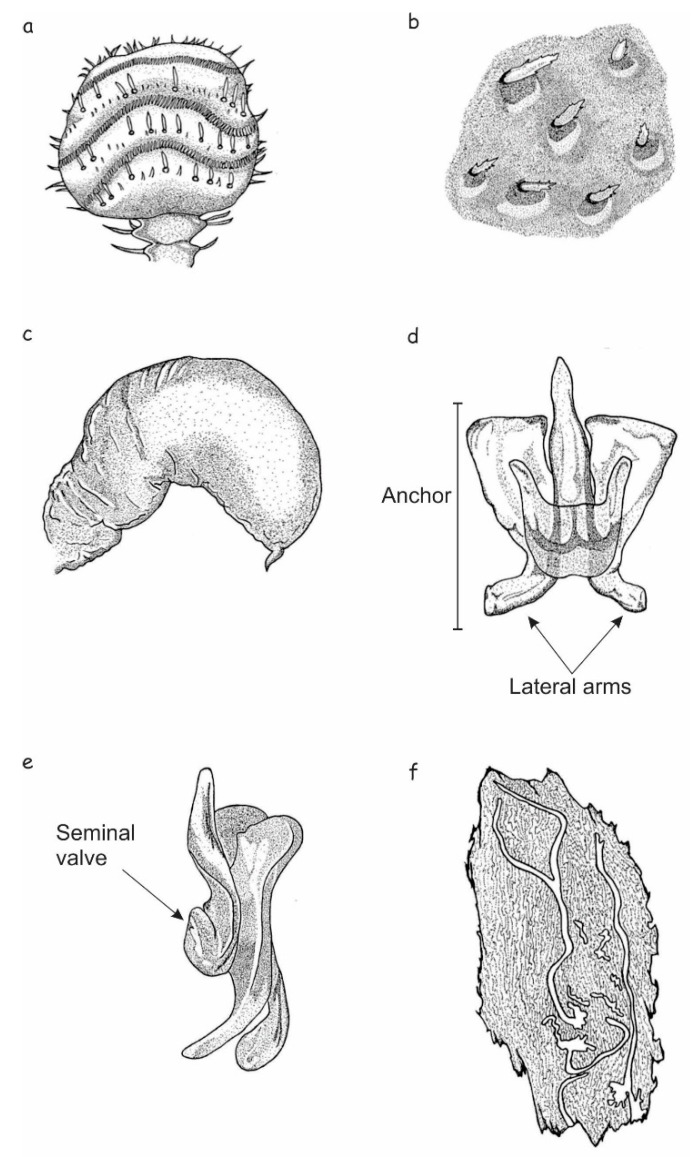
*Dendroctonus barberi*: (**a**) Antennal club, (**b**) elytral sculpturing, (**c**) spermatheca, (**d**,**e**) seminal rod in dorsal and lateral view, (**f**) parental tunnels in host bark.

Total length, 2.6–4.7 mm ($\bar{X}$ = 3.91 mm); head black to dark brown; pronotum with somewhat lighter color, elytra same color as pronotum (Figure 9a,b); chromosome formula = 5AA + neo-XX. Head: Similar to *D*. *brevicomis* females, except differing somewhat in length (0.20–0.53 mm, $\bar{X}$ = 0.36 mm) and epistomal process width (0.17–0.52 mm, $\bar{X}$ = 0.34 mm); arms of epistomal process slightly elevated, oblique, and about 10° from horizontal (Figure 3d). Length of epistomal brush 0.13–0.26 mm ($\bar{X}$ = 0.20 mm). Pubescence of head from brown to orange, densely distributed around frons and less abundant on area immediately above epistomal process (Figure 9d,e). Antenna: General organization similar to *D*. *brevicomis*, but with three very curved bands of sensillae (Figure 10a). Pronotum: Similar to *D*. *brevicomis* female specimens, except in length 0.67–1.24 mm ($\bar{X}$ = 1.05 mm) and width 1.07–1.79 mm ($\bar{X}$ = 1.51 mm). As with *D. brevicomis* female, elevated callus on present on anterior margin of pronotum (Figure 9c). Elytra: Similar to *D*. *brevicomis* female specimens except in length, 1.74–3.0 mm ($\bar{X}$ = 2.5 mm), ~1.6 times longer than wide and 2.3 times longer than pronotum; sides straight on proximal two-thirds, rounded behind; elytral declivity convex with striae strongly impressed (Figure 9f–h); punctures with a diameter of striae I 17.9–23.7 μm ($\bar{X}$ = 21.0 μm) surface between smooth punctures, punctures spaced 18.7–43.3 μm ($\bar{X}$ = 33.6 μm) apart; surface of interstriae with weakly or strongly marked rugged, small punctures and crenulations. Punctures on declivital interstriae well defined and smaller than those of striae (Figure 9f–h); crenulations in interstriae of variable size, more defined and abundant in anterior area of elytral disc and scarce or absent towards posterior of elytral disc (Figure 9h and Figure 10b); setae in interstriae I–III of elytral declivity only one size class with yellow to amber color in mounted specimens (Figure 9f); setae on elytral disc and declivity with sawed edge. Length of setae is usually less than the distance between them. Diameter of the punctures in the striae II on the elytral declivity 0.017–0.023 ($\bar{X}$ = 0.020 μm), distance between punctures in the striae II 0.018–0.046 ($\bar{X}$ = 0.03 μm), number of pubescences in the striae II 23–47 ($\bar{X}$ = 34), length of pubescences in the elytral declivity 0.026–0.040 ($\bar{X}$ = 0.033 μm), width of pubescences in the elytral declivity 0.0047–0.0067 ($\bar{X}$ = 0.005 μm), width of the intestria II in the distal 0.052–0.102 ($\bar{X}$ = 0.079 μm), an proximal region 0.040–0.102 ($\bar{X}$ = 0.072 μm). Genitalia: Spermathecae reniform and divided into a proximal nodulus and distal cornu; nodulus surface transversely covered by striations over nearly its entirety, becoming confused in distal region of nodulus; striations in the proximal region of nodulus not aggregate; the anterior and posterior edges of nodulus are rounded, and the cornu is widened and ovate distally (Figure 10c).

#### 4.3.6. Description of the Male

Total length, 2.5–4.7 mm ($\bar{X}$ = 3.77); 2.6 times longer than wide; body color similar to female; chromosome formula = 5AA + XY. Head: Similar to female except in length (0.25–0.54 mm, $\bar{X}$ = 0.4 mm). Frons with a pair of evident frontal tubercles, one on each side of the middle furrow, that may or may not fused (Figure 9g); width of epistomal process 0.19–0.51 mm ($\bar{X}$ = 0.36 mm), 0.44 times the distance between eyes; arms of epistomal process strongly elevated, oblique and about 10° from horizontal; underside with a dense brush of setae 0.13–0.20 mm ($\bar{X}$ = 0.2 mm); distance between eyes 0.58–0.94 mm ($\bar{X}$ = 0.80 mm), width of eyes 0.18–0.27 mm ($\bar{X}$ = 0.22 mm), length of eyes 0.33–0.75 mm ($\bar{X}$ = 0.48 mm). Antenna: Similar to the female (Figure 10a). Pronotum: Similar to females, except in length (0.64–1.15 mm, $\bar{X}$ = 0.97mm) and width (1.02–1.72 mm, $\bar{X}$ = 1.42); widest on posterior third, anterior region slightly constricted. Lacking pronotal callus of female. Elytra: Similar to female specimens, except in the length (1.69–3.04 mm, $\bar{X}$ = 2.39 mm), 1.6 times longer than wide, 2.7 times longer than pronotum; sides parallel on the proximal sides, rather broadly rounded in the distal region; declivity convex with striae strongly impressed (Figure 9f–h and Figure 10b). Genitalia: Seminal rod with the same elements as *D*. *brevicomis* male specimens; this structure displays two curved lateral arms in dorsal view, which are joined to the seminal rod base.

Dorsal process of the seminal rod (seminal rod body) enclosed within a triangular plate (Figure 10d,e). Seminal rod entire and slightly longer in the seminal rod body than *D*. *brevicomis* with a seminal rod body that ends in an apex; in dorsal view, length of the seminal rod body from four to five times the length of seminal valve (Figure 10d); lateral arms of seminal rod curved and not fused. The lateral view of the seminal rod is thickened distally; seminal valve of the seminal rod body without a projection (Figure 10e).

Gallery. Transversely winding (Figure 10f) [12].

Distribution. This species occurs in the USA and Mexico: From Utah, Nevada, Colorado, Arizona, New Mexico, western Texas, Chihuahua, and Durango.

## 5. Final Considerations

Currently, *D. brevicomis* is geographically isolated from *D. barberi* by the Great Basin, and Mojave and Sonoran deserts in North America. Based on the results, we hypothesize that the distribution of *D. brevicomis* occurs from British Columbia, Canada throughout western USA to southern California [12,15], including Idaho and Montana; whereas *D barberi* occurs in Nevada, Utah, Colorado, Arizona, New Mexico, and Texas, USA, and in Chihuahua and Durango, Mexico. This geographic partitioning of the insect taxa reflects that of their host species [48]: The *D. brevicomis* range coincides with that of host species *P. ponderosa*, *P. benthamiana*, and *P. coulteri*; whereas *D. barberi* occurs with hosts *P*. *arizonica* var. *stormiae*, *P. brachyptera*, *P. scopulorum*, and *P. arizonica* Shaw and *P. engelmannii* Carrier Mexico [2,48].

Lastly, an unexpected finding in our study was the strong morphological differentiation of SMOR, a population of *D. brevicomis* occurring only in Mexico (Coahuila, Nuevo León, and Tamaulipas states). Morphometric differences of the spermatheca and seminal rod in dorsal view were more pronounced between SMOR and both *D. barberi* (East-SMOC) and *D. brevicomis* (West) than differences between these latter two groups. Nonetheless, we were unable to identify diagnostic external characters for differentiating SMOR from East-SMOC. Thus, we conclude that the SMOR specimens might be a distinct species from *D. barberi* and *D. brevicomis*. However, molecular and additional studies are necessary before a formal description of this taxon can be justified. For now, SMOR specimens should be considered as *D. barberi*.

## 6. Conclusions

In this study, we provide lineal and geometric morphometric evidence to reinstate *Dendroctonus barberi*, and remove it from synonymy with *D. brevicomis*. The pubescence length on the elytral declivity, the genitalia shape both female and male in dorsal view, antennae shape and curvature of the three sensory bands, and the parental tunnels shape are robust characters for segregation of these species (Figure 8 and Figure 10). In addition, molecular and chemical ecology data published and in progress support this decision. On the other hand, differences in some quantitative and qualitative characters, as well as the spermatheca and seminal rod shape of SMOR specimens, suggest that these populations might be a different species from *D. barberi* and *D. brevicomis*, which should be confirmed in future studies.

## Supplementary Materials

The following are available online at https://www.mdpi.com/2075-4450/10/11/377/s1: Table S1. Localities of *D*. *brevicomis* samples analyzed; Table S2. Qualitative morphological characters analyzed among geographical groups; Table S3. ANOVA and Tukey test results of continuous morphological characters among females and males, males alone, and females alone of different geographical groups; Figure S1. Landmarks and semilandmarks in antennae, spermathecae, and seminal rod in dorsal and lateral view; Figure S2. Deformation grids of antennae, spermatheca, and seminal rod in dorsal and lateral view of *D. brevicomis* specimens.
