# Supplementary material for: Cryptic Species Discrimination in Western Pine Beetle, Dendroctonus brevicomis LeConte (Curculionidae: Scolytinae), Based on Morphological Characters and Geometric Morphometrics
Source: Insects. 2019 Oct 30;10(11):377. doi: 10.3390/insects10110377 (PMC6920968; doi:10.3390/insects10110377)

Table S1. Localities acronyms, country, state, locality and geographical coordinates of *D. brevicomis* samples analyzed.

Acronym	Country, state and localities	Longitude	Latitude	Number of specimens: n (morphological characters)	antennae	spermathecae	Seminal rod: Dorsal view.	Seminal rod: Lateral view
**CCBA**	Canada, British Columbia, Aspen Grove	49°55'49"	120°37'47	30	18	17	7	10
**EUPW**	United States, Washington, Pullman	46°43'59"	117°9'60"	5	5	2	3	3
**EUDW**	United States, Washington, Dayton	46°16'40"	117°48'52"	5	5	2	3	3
**EBMW**	United States, Washington, Blue Mountains.	46°21'31"	117°41'3"	5	5	3	2	2
**ESPW**	United States, Washington, Signal peak	46°13' 37"	121°8'15"	5	5	3	2	2
**EUFW**	United States, Washington, Ferry Co.	48°28'17"	118°29'50"	7	7	4	3	3
**EUOW**	United States, Washington, Okanogan.	48°21'40"	119°35'6"	7	7	3	4	4
**EYCW**	United States, Washington, Co.	47°45'3"	120°44'24"	5	5	2	3	3
**EUSW**	United States, Washington, Sherman Creek Wildlife Management Area	48°36'17"	118°10'60"	30	17	8	13	14
**EUPO**	United States, Oregon, Prineville	44°17'59"	120°50'4"	5	5	2	3	3
**EDCO**	United States, Oregon, Deschutes	43°49'33"	121°15'40"	5	5	2	3	3
**EUCO**	United States, Oregon, Corvalis	44°33'50"	123°15'43"	5	5	3	2	2
**EUSO**	United States, Oregon, Sister's	44°17'27"	121°32'57"	5	5	3	2	2
**EKFO**	United States, Oregon, K. Falls	42°13'29"	121°46'54"	5	1	2	3	3
**EWNO**	United States, Oregon, Wallowa National Forest	45°19'60"	117°0'5"	9	9	5	*	*
**EUSCO**	United States, Oregon, Spring Creek	42°40'46"	121°53'32"	22	14	2	8	21
**ESDC**	United States, California, San Diego Co.	32°42'54"	117°9'39"	10	*	3	7	7
**EMSC**	United States, California, Mountain Shasta	41°16'45"	122°14'54"	30	19	5	7	11
**EUMCC**	United States, California, Mariposa Co.	37°29'24"	119°57'59"	5	*	3	2	2
**EOMC**	United States, California, Oakhurst Madera Co.	37°15'7"	119°41'46"	5	*	2	3	3
**EUSJC**	United States, California, San Jacinto	33°47'23"	116°56'53"	8	3	3	*	3
**EMRSI**	United States, Idaho, Magruder R.S.	45°42'8"	114°38'11"	5	2	2	3	3
**ECDAI**	United States, Idaho, Cœur d'Alene	47°48'38"	116°24'10"	5	3	3	2	2
**EULI**	United States, Idaho, Lowman	44°4'51"	115°37'7"	5	1	*	5	5
**ESRCI**	United States, Idaho, Snake River Canyon	45°34'55"	116°28'53"	5	5	2	3	3
**EICI**	United States, Idaho, Idaho City	43°49'42"	115°50'4"	5	2	2	3	3
**EATI**	United States, Idaho, Athol	47°56'52"	116°42'29"	5	1	3	*	*

**EUTI**	United States, Idaho, Tamarack	44°57'18"	116°23'8"	5	3	2	2	2
**ESKI**	United States, Idaho, Kootenai Co.	47°45'24"	116°37'20"	25	3	*	7	8
**EKKI**	United States, Idaho, Kooskia	46° 8'41"	115°58'41"	5	*	*	*	*
**EWCI**	United States, Idaho, Willow Creek Campground	43°38'39"	115°45'14"	5	*	*	*	*
**EUMI**	United States, Idaho, Moscow	46°48'18"	116°52'6"	5	*	*	*	*
**EBNFI**	United States, Idaho, Boise National Forest	43°48'45"	115°33'45"	9	1	2	*	*
**EULM**	United States, Montana, Lolo National Forest	47°34'60"	115°35'4"	5	1	*	1	1
**EUDM**	United States, Montana, Drummond	46°39'58"	113°8'49"	5	1	*	1	*
**ESMN**	United States, Nevada, Springs Mountains	36°17′53″	115°39'50"	5	1	4	1	1
**EPVU**	United States, Utah, Pine Valley	37°4′33″	113°34'52"	5	*	3	2	*
**EOCU**	United States, Utah, Oak Grove Campground	37°18'59"	113°27'12"	13	*	*	4	4
**EFUT**	United States, Utah, Fishlake National Forest	38°40'54"	112°20'2"	5	5	4	*	*
**ELSU**	United States, Utah, La Sal National Forest	39°16'59"	111°25'29"	8	4	7	*	*
**EACC**	United States, Colorado, Archuleta Co.	37°9'38"	107°0'24"	5	6	*	5	6
**EMCA**	United States, Arizona, Moqui Camp	34°35'46"	111°11'55"	5	2	2	*	1
**EUTA**	United States, Arizona, Tuyasan Ranger Station	35°14'14"	112°12'55"	5	1	*	3	3
**EUYA**	United States, Arizona, Young	34°5'38"	110°56'11"	5	3	*	2	2
**EPNFA**	United States, Arizona, Prescott National Forest	34°34'36"	112°34'20"	20	*	5	5	5
**EUNFA**	United States, Arizona, North Flagstaff	35°11'4"	111°38'2"	11	8	2	2	3
**ELNFN**	United States, New Mexico, Lincoln National Forest	32°50'2"	105°41'49″	16	4	4	3	2
**EUCM**	United States, New Mexico, Cloudcroft	32°56'39"	105°44'59"	5	5	*	2	1
**EUSNN**	United States, New Mexico, Santa Fe National Forest	35°56'3"	105°39'49"	30	11	6	5	10
**EUMCT**	United States, Texas, Madera Canyon	30°55'43"	103°48'49"	28	31	8	11	13
**CGRA**	Mexico, Chihuahua, Nicolas Bravo	28°38'45.06"	106°1'46.63"	5	3	2	*	2
**CGEC**	Mexico, Chihuahua, Guachochi, Rincones del Aguajito	26°58'12.2"	107°06'12.1"	5	2	3	*	2
**CGA**	Mexico, Chihuahua, Guachochi, Ejido Corralitos	26°54'40.0"	106°58'38.8"	5	4	1	2	*
**CGRP**	Mexico, Chihuahua, La Angostura	26°56'56.4"	107°06'00.2"	5	1	1	3	*
**CGMA**	Mexico, Chihuahua, Guachochi Rocheachi, Pesachi	27°4'55.91"	107°12'2.50"	5	1	2	2	1
**CGFL**	Mexico, Chihuahua, Guachochi, Mesa del Aguaje	27°05' 38.9"	107°15'18.4"	5	2	*	1	1
**CGTH**	Mexico, Chihuahua, Guachochi, Fraccionamiento A, lote 1, la Lobera	26°53'01.9"	107°06'43.3"	5	2	3	1	1

**CGTS**	Mexico, Chihuahua, Guachochi, Ejido Tatahuichui Hueleyvo	27°15'33.8"	107°22'52.9"	5	1	3	1	2
**CGAT**	Mexico, Chihuahua, Guachochi, Tonachi Sibarichi	26°56'46.9"	107°15'28.5"	5	2	2	*	2
**CGAP**	Mexico, Chihuahua, Guachochi, Ajolotes Telesforo	26°52'45.0"	107°02'28.8"	5	3	*	*	2
**CGF4**	Mexico, Chihuahua, Guachochi, Aboreachi potrero Eusebio	27°06'26.4"	107°20'14.9"	5	4	1	1	*
**CGSA**	Mexico, Chihuahua, Guachochi, Fraccionamiento 4, Rancho Roque	26°43'50.2"	107°10'32.8"	5	2	1	2	1
**CGP**	Mexico, Chihuahua Guachochi, Samachique Aserradero	27°17'49.5"	107°32'51.3"	5	2	2	2	1
**CGVL**	Mexico, Chihuahua, Guachochi, El Peñasco	26°53'19.3"	107°05'41.3"	5	1	3	*	2
**CGLS**	Mexico, Chihuahua, Guachochi, Valle de Lobos	26°55'46.6"	107°08'23.2"	5	1	*	4	*
**CGRE**	Mexico, Chihuahua, Guachochi, Ejido La Soledad	26°56'54.9"	106°59'36.7"	5	2	1	2	1
**CGL2**	Mexico, Chihuahua, Guachochi, Rancho La Esperanza	26°52'39.9"	107°11'32.6"	5	1	1	2	1
**CGAZ**	Mexico, Chihuahua, Guachochi, Lote 2	26°52'19.6"	107°08'37.2"	5	1	2	2	1
**CGT**	Mexico, Chihuahua, Guachochi, Ejido Agua Zarca.	26°49'15.1"	107°08'11.1"	5	3	2	*	1
**CGT2**	Mexico, Chihuahua, Guachochi, El Tascate	26°47'13.7"	107°07'33.8"	5	2	*	1	1
**CGPE**	Mexico, Chihuahua, Guachochi, Caborachi	26°49'23.1"	106°55'38.2"	5	1	3	1	2
**CML**	Mexico, Chihuahua, Guachochi, Patio Elio Acosta	26°51'37.46"	107°5'11.52"	5	1	3	1	2
**CMG**	Mexico, Chihuahua, Madera	29°26'03"	108°11'38"	5	2	1	2	2
**DSO**	Mexico, Durango, Ejido Otinapa y San Carlos	24°02'25.8"	105°04'23.3"	5	1	1	2	1
**DSC**	Mexico, Durango, PP. Chavarria y Municipio San Dimas	24°22'8.3"	105°32'48.2"	5	*	1	2	1
**DPE**	Mexico, Durango, Parque Ecológico Tecúan	23°56'59.91"	105° 3'0.02"	5	2	*	1	1
**DAM**	Mexico, Durango, Ejido Altares, pareja "Mesa del Cristo"	23°56'59.91"	105° 3'0.02"	5	2	1	1	1
**MACH**	Mexico, Coahuila, Sierra de Arteaga.	25°12′49.7″	100°14′59″	5	*	1	1	1
**MNCP**	Mexico, Nuevo León, Cerro el Potosí.	24°52′2″	100°13′52″	30	17	5	6	8
**MLGY**	Mexico, Nuevo León, Linares Galena, la "Y".	24°46′45″	100°2′42″	30	9	10	4	8
**MGPP**	Mexico, Nuevo León, Galeana Puerto Pastores.	24°46′42″	100°2′1″	28	6	5	2	15
**GFT**	Mexico, Tamaulipas, Ejido Gómez Farías.	22°52′18″	99°2′44″	5	*	2	1	1

Figure S1. Landmarks and semilandmarks configurations in: a) antennae, b) spermathecae, c-d) seminal rod in dorsal and lateral view.

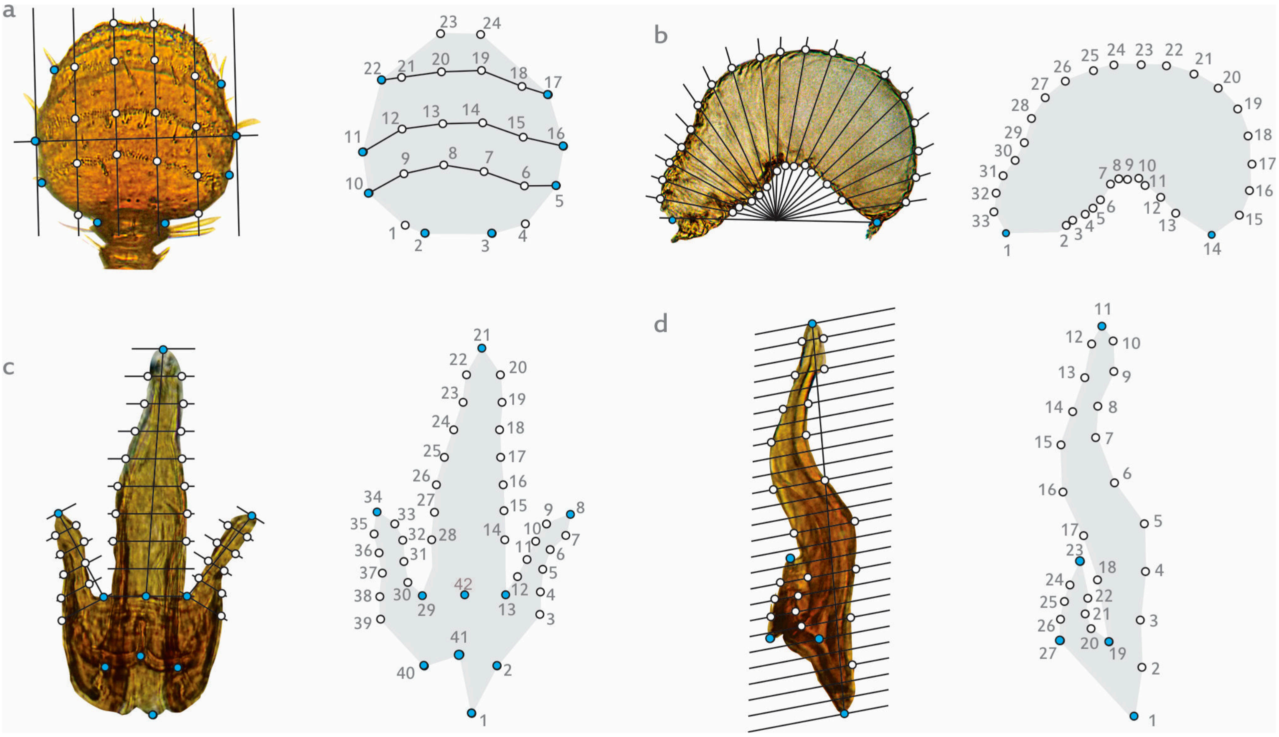

Table S2. Qualitative morphological characters analyzed among geographical groups. **FS** – Frons sculpture, **DE** - Degree of elevation in the epistomal process, **ES** - Epicranium surface, **SPD** - Size of the pubescences in the elytral declivity, **RSP** - Pubescen in the striae of the elytral declivity, **SED** - Striae on the elytral declivity, **PNCS** – Proportion of the nodulus covered by striae, **CS** - Cornu Shape, **PC** - Protuberance of cornu,

Character/Geographical group	**W**	**East-SMOC**	**SMOR**	**W**	**East-SMOC**	**SMOR**	**Chi2 (p ≤ 0.05)**
**FS**	Scarce			Abundant			
n	135	112	93	75	86	26	*P* = 0.0005
%	39.84%	32.95%	27.20%	40.10%	46%	13.90%	
**DE**	Elevated			unelevated			
n	50	33	17	159	166	102	No
%	50.00%	33.00%	17.00%	37.30%	38.83%	23.85%	
**ES**	Smooth			Rough			
n	81	95	56	123	112	60	No
%	35.00%	41%	24.17%	41.81%	38%	20.25%	
**SPD**	Variable			Uniform			
n	210	0	0	0	198	119	*P* = 0.0005
%	100%	0%	0%	0%	63%	37%	
**RSP**	Thin			Thick			
n	125	173	119	85	25	0	*P* = 0.0005
%	30.00%	41.56%	28%	77.38%	22.61%	0	
**SED**	Impressed			Poorly or not impressed			
n	179	119	1	31	79	118	*P* = 0.0005
%	59.82%	39.73%	0.43%	13.71%	34.85%	51.42%	
**PNCS**	Half			Complete			
n	18	37	24	0	108	16	
%	23.33%	47%	30%	0	87.23%	12.76%	*P* = 0.0005
**♀ CS**	Oval			Rounded			
n	38	95	23	6	35	6	No
%	24.07%	61.11%	15%	12.50%	75.00%	13%	
**♀ PC**	Absent			Present			
n	3	25	0	36	114	25	No
**%**	9.09%	90.90%	0	20.28%	65.25%	14%	

The percentaje represents el individual numbers in each geographical region that have one or the other state of character.

Table S3. ANOVA and Tukey test results of continuous morphological characters among females and males, alone males, and alone females from D. brevicomis of different geographical groups. **FTL**-Frontal tubercles length, **DFT**-Distance between frontal tubercles, **EBL**-Epistomal brush length, **EPW**-Epistomal process width, **EW**-Eye width, **EL**-Eye length, **DBE**-Distance between the eyes, **HPL**-Head-Pronotum length, **PL**-Pronotum Length, **PW**-Pronotum width, **EYL**-Elytra length, **AL**-Abdominal length, **LMM**-Length of the midline of the metathorax.

Females-males

Character	WEST	EAST-SMOC	SMOR	*P* < 0.05
**EBL**	219.45 ± 63.57 **a**	206.57 ± 27.62 **a, b**	224.01 ± 33.30 **a, c**	*
**EPW**	371.09 ± 71.45 **a**	351.13 ± 68.69 **b, c**	387.52 ± 77.07 **a, d**	*
**EW**	233.20 ± 61.83	224.43 ± 20.51	235.21 ± 27.00	No
**EL**	514.4 ± 73.02 **a**	492.58 ± 53.41 **b**	535.71 ± 84.09 **c**	*
**DBE**	840.92 ± 99.32 **a**	819.09 ± 82.61 **a, b**	847.25 ± 108.05 **a, c**	*
**HPL**	1443.38 ± 198.31 **a**	1402.98 ± 165.89 **a, c**	1499.78 ± 223.80 b, **d**	*
**PL**	1027.29 ± 143.87 **a**	1022.16 ± 118.32 **a, b**	1066.19 ± 155.95 **a, c**	*
**PW**	1510.63 ± 188.41 **a**	1479.45 ± 158.11 **a, b**	1552.17 ± 203.75 **a, c**	*
**EYL**	2433.52 ± 327.43 **a**	2457.90 ± 273.05 **a, c**	2569.81 ± 310.53 **b, d**	*
**AL**	1237.58 ± 158.52 **a**	1205.79 ± 133.11 **a, c**	1264.28 ± 156.24 **b, d**	*
**LMM**	631.95 ± 115.48 **a**	649.19 ± 78.31 **a, c**	735.49 ± 108.75 **b, d**	*

*Significant

Females

Character	WEST	EAST-SMOC	SMOR	*P* < 0.05
**EBL**	221.50 ± 80.39	207.25 ± 25.31	221.66 ± 28.22	No
**EPW**	365.36 ± 66.55	340.42 ± 70.19	363.66 ± 59.06	No
**EW**	236.19 ± 73.85	227.55 ± 20.69	238.33 ± 25.22	No
**EL**	523.0692 ± 77.13 **a**	497.04 ± 52.57 **a, c**	537.33 ± 101.59 **a, d**	*
**DBE**	843.93 ± 102.78	829.13 ± 85.25	861.66 ± 102.31	No
**HPL**	1466.05 ± 196.69 **a**	1412.54 ± 166.30 **a, c**	1556.62 ± 221.29 **b, d**	*
**PL**	1060.03 ± 153.96 **a**	1051.79 ± 115.79 **a, c**	1132.8 ± 148.59 **b, d**	*
**PW**	1538.37 ± 203.37 **a**	1514.65 ± 159.08 **a, b**	1606.4 ± 190.97 **a,c**	*
**EYL**	2480.24 ± 361.97 **a**	2502.87 ± 276.90 **a, c**	2664.53 ± 283.79 **b, d**	*
**AL**	1255.50 ± 175.84 **a**	1232.07 ± 135.88 **a, b**	1306.33 ± 144.40 **a, c**	*
**LMM**	643.98 ± 126.61 **a**	661.30 ± 78.76 **a, c**	761.66 ± 106.54 **b, d**	*

*Significant

Males

Character	WEST	EAST-SMOC	SMOR	*P* < 0.05
**FTL**	88.16 ± 19.43	84.79 ± 21.93	80.86 ± 18.29	No
**DFT**	225.95 ± 38.54 **a**	206.57 ± 35.60 **b**	211.95 ± 36.41 **a**	*

**EBL**	218.49 ± 31.38 **a**	204.42 ± 30.35 **a, c**	226.30 ± 37.39 **b**, d	*
**EPW**	382.58 ± 75.72 **a**	362.77 ± 64.86 **a, b**	410.86 ± 85.70 **a, c**	*
**EW**	229.28 ± 43.37	220.6824 ± 19.82	232.17 ± 28.59	No
**EL**	504.85 ± 64.99 **a**	485.61 ± 56.73 **a, c**	534.13 ± 63.59 **b, d**	*
**DBE**	836.66 ± 88.81	802.56 ± 81.99	833.15 ± 112.72	No
**HPL**	1421.44 ± 196.71	1383.10 ± 167.44	1444.17 ± 214.24	No
**PL**	988.08 ± 115.93	976.27 ± 112.15	1001.04 ± 135.20	No
**PW**	1481.03 ± 156.68 **a**	1424.082 ± 149.01 **a, b**	1499.13 ± 203.83 **a, c**	*
**EYL**	2371.63 ± 261.33	2393.88 ± 263.04	2470.69 ± 309.35	No
**AL**	1216.35 ± 128.10 **a**	1167.44 ± 121.67 **a, b**	1223.15 ± 157.91 **a, c**	*
**LMM**	616.38 ± 96.09 **a**	629.89 ± 78.74 **a**	709.89 ± 105.83 **b**	*

*Significant

Figure S2. Deformation grids of antennae, spermatheca, and seminal rod in dorsal and lateral view of *D. brevicomis* specimens.

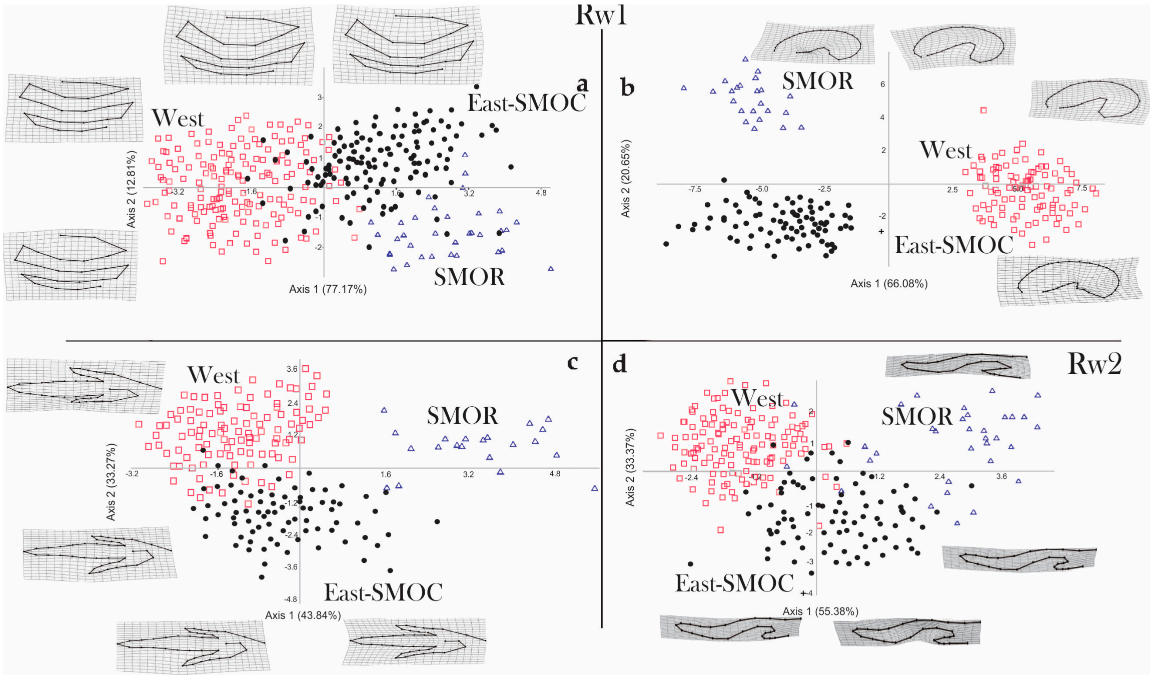

Supplement: Supplementary file 1 [file insects-10-00377-s001.pdf]
